# Acoustic Coupling between Resonator Tubes in Quartz-Enhanced Photoacoustic Spectrophones Employing a Large Prong Spacing Tuning Fork

**DOI:** 10.3390/s19194109

**Published:** 2019-09-23

**Authors:** Stefano Dello Russo, Marilena Giglio, Angelo Sampaolo, Pietro Patimisco, Giansergio Menduni, Hongpeng Wu, Lei Dong, Vittorio M. N. Passaro, Vincenzo Spagnolo

**Affiliations:** 1 PolySense Lab, Physics Department, Politecnico and University of Bari, CNR-IFN, I-70100 Bari, Italy; stefano.dellorusso@uniba.it (S.D.R.); marilena.giglio@poliba.it (M.G.); angelo.sampaolo@poliba.it (A.S.); pietro.patimisco@poliba.it (P.P.); giansergio.menduni@poliba.it (G.M.); 2 Photonics Research Group, Dipartimento di Ingegneria Elettrica e dell’informazione, Politecnico of Bari, I-70126 Bari, Italy; Vittorio.passaro@poliba.it; 3 State Key Laboratory of Quantum Optics and Quantum Optics Devices, Institute of Laser Spectroscopy, Shanxi University, Taiyuan 030006, China; wuhp@sxu.edu.cn

**Keywords:** quartz tuning fork, resonator tubes, spectrophone, photoacoustic spectroscopy, gas sensing

## Abstract

A theoretical model describing the acoustic coupling between two resonator tubes in spectrophones exploiting custom-made quartz tuning forks (QTFs) is proposed. The model is based on an open-end correction to predict the optimal tube length. A calculation of the sound field distribution from one tube exit allowed for the estimation of the optimal radius as a function of the QTF prong spacing and the sound wavelength. The theoretical predictions have been confirmed using experimental studies employing a custom QTF with a fundamental flexural mode resonance frequency of 15.8 kHz and a quality factor of 15,000 at atmospheric pressure. The spacing between the two prongs was 1.5 mm. Spectrophones mounting this QTF were implemented for the quartz-enhanced photoacoustic detection of water vapor in air in the mid-infrared spectral range.

## 1. Introduction

Quartz-enhanced photoacoustic spectroscopy (QEPAS) is a spectroscopic technique aimed at trace gas detection. This technique exploits the photoacoustic effect that occurs in an absorbing gas, i.e., the absorption of the modulated light resonant with a selected gas absorption feature and the subsequent generation of sound waves with an intensity proportional to the target gas concentration [1,2]. In QEPAS, sound waves with a frequency *f_0_* are detected using a resonant quartz tuning fork (QTF). Sound waves are generated between the QTF prongs and deflect the two prongs in opposite directions in the QTF plane, exciting QTF piezoelectrically-active, anti-symmetrical flexural modes. The QTF is not used as a standalone sound wave detector: it is acoustically coupled with a pair (or a single) of resonator tubes located on both sides (or between the prongs) of the QTF [3,4,5]. Resonator tubes act as an organ pipe acoustic resonator and can enhance the intensity of the acoustic field between the QTF prongs up to 60 times [6]. The QTF coupled with a pair of resonator tubes constitutes the QEPAS spectrophone and represents the detection unit of sound waves in a gas. 

The impact of the spectrophone design parameters on QEPAS performance was investigated in different experimental studies [3,5]. For a selected QTF, the geometric parameters influencing the sensor performance are the internal diameter (*ID*) and the length of the two tubes *l*. The length of the two tubes is correlated with the sound wavelength *λ = v_s_/f_0_*, where *v_s_* is the sound speed in the sample gas mixtureair. If the gap between the tubes is neglected, each tube forms a half-wave resonator with the QTF placed on the antinode point of the sound field. Experimental studies have shown that the optimal tube length is less than *λ/2* and this effect has been ascribed to the interaction between the two resonator tubes and to their acoustic coupling with the QTF [5]. The choice of the optimal *ID* has been so far related only to the QTF prongs’ spacing. When the tube diameter is comparable with the prongs’ spacing, the two tubes are acoustically coupled to each other and the interaction with the QTF becomes negligible, leading to a low QTF signal. When the tube diameter is larger than the prongs’ spacing, the QTF strongly interact oscillates with the sound wave generated in the two tubes and a good coupling between the QTF and dual-tube system can be achieved. Several studies showed that tubes with a length of 4.4 mm and internal diameter of 0.6 mm are the best choice for the standard 32.7 kHz QTF [3]. A finite element modeling simulation software based on COMSOL Multiphysics has been used to predict the optimal tube length for a dual-tube system coupled with a QTF having a prong gap as narrow as *s* = 200 µm [7]. An extensive experimental investigation has been performed to study the optimal tube parameters when a QTF with a resonance frequency of 30.72 kHz is used in QEPAS sensors [8,9]. The first implementation of microresonator tubes with a custom-made QTF was reported in Wu et al. [10]. The tuning fork employed had a prong spacing *s* = 0.8 mm and a resonant frequency of 7205 Hz. The used pair of tubes had an inner diameter of 1.3 mm. These studies revealed that for a tube length of 23 mm, the signal-to-noise ratio was enhanced by a factor of ≈40 compared to that measured for the bare QTF. Other spectrophones have been assembled with custom-made QTFs: all obtained results showed that the best configurations are obtained when the length of each tube is slightly lower than half of the sound wavelength and the internal diameter is larger than the prongs spacing [11,12,13,14,15]. So far, there is no way to predict in advance the best resonator geometrical parameters and they must be determined experimentally. Spectrophones with QTFs having a prong spacing larger than 1.0 mm have not yet been adopted in a QEPAS sensor, though this could be advantageous in terms of noise reduction. Indeed, the detection sensitivity of a QEPAS sensor is limited by the fraction of laser light hitting the prong surface. The absorption of light from the quartz crystal gives rise to a photothermal noise contribution that can be several times stronger than the ultimate noise level reachable (typically the QTF Johnson noise). When operating with a small prong spacing, to limit the photothermal noise contribution, laser sources with a high spatial beam quality are required, or alternately, spatial filters (pinhole) or modal filters should be used to filter out higher order modes, but this can lead to a sensitive reduction of the available optical power. QTFs with a large prong spacing can overcome these limitations, also leading to an easy optical alignment.

In this work, we propose a theoretical model capable of predicting both the optimal internal diameter and length of the tubes of a QEPAS spectrophone as a function of the QTF prongs geometry and spacing. The theoretical model is based on the open-end correction of resonators, the divergence of the sound field exiting one tube, and its acoustic coupling with the other tube. To test and validate the model, we designed and realized a set of QEPAS spectrophones composed of a 15.8 kHz QTF with a prong spacing *s* = 1.5 mm and 48 pairs of resonator tubes differing in length and internal diameter. Each spectrophone was positioned in an acoustic detection module, including a gas cell, two optical windows, and gas-in and -out connectors, for QEPAS sensing. The gas target was water vapor and the selected absorption line falls in the mid-infrared spectral range. The experimental data have been compared with theoretical simulation.

## 2. Resonator Tubes 

A resonator tube is an open-ended, rigid circular pipe with a negligible wall thickness. When a sound wave generated by a source located close to one end of the resonators propagates through a resonator tube, a standing wave vibrational pattern is created within the resonator when multiple reflected waves from the ends of the resonator constructively interfere with the incident wave, in a way that makes specific points along the resonator appear to be standing. If the resonator tube diameter is small compared to the sound wavelength, the excited sound field varies only along the resonator length, resulting in a one-dimensional acoustic field. Hence, a resonator tube can be considered as a one-dimensional acoustic resonator. In this approximation, a nearly complete reflection of a dominant mode sound wave occurs at the open end of the resonator. Hence, the standing wave pattern’s antinodes should correspond to the open ends of microresonator. Actually, the antinode of a standing sound wave in a resonator with an open end is located slightly outside the end point, at a distance called the open-end correction (OEC). The OEC can be explained by considering the effect of the air surrounding the resonator on the pressure field inside the tube. The mismatch between the one-dimensional acoustic field inside the resonator and the three-dimensional field radiated by the open end outside the resonator causes the pressure field outside the tube to oscillate with the one inside. Therefore, differing from the free-edge boundary conditions, the air inside the tube is affected by the reaction force from the air outside. As a result, the antinode appears a little away from the end of the tube. Ogawa and Kaneko [16] estimated the open-end correction value *Δl* as being:

(1) $$\Delta l=\;\;\frac{8a\;\;}{3\pi }$$

where *a* is the radius of the acoustic resonator. The OEC should be added to the length of the tube. Therefore, an open–end resonator should have resonances when the sum of the resonator length and the OEC is equal to an integer multiple half the wavelength. Thereby, the corresponding resonance frequencies can be obtained from the following expression:

(2) $$f_{n}=\frac{nv_{s}}{2\left(l+2\Delta l\right)}$$

where *n* = 1, 2, 3, … identifies the mode orders of standing waves, *v_s_* is the sound velocity, and a factor of 2 has been taken into account to consider the two openings of each resonator. By using Equations (1) and (2), the effective length of the resonators for the dominant mode (n = 1) can be estimated as:

(3) $$l=\;\frac{v_{s}}{2f_{0}}-\frac{16a}{3\pi }\;$$

When the propagating waves approach the resonator end, part of the incident waves is back-reflected into the resonator, while the transmitted wave propagates outside the resonator. Levine, H.; Schwinger [17] determined the amplitude and phase of the back-reflected wave and the amplitude of the diverging spherical wave at a large distance from the resonator end. By choosing the end of the resonator as a reference plane, the distribution of the sound emitted by an isotropically radiating point source at an angle *θ* measured from the axis of the resonator, *G*(*ka,θ*)*,* is described using the function:

(4) $$G\left(ka,\theta \right)=\frac{4}{\pi sin^{2}\theta }\frac{J_{1}\left(kasin\theta \right)}{\sqrt{J_{1}{\left(kasin\theta \right)}^{2}+{\left[N_{1}\left(kasin\theta \right)\right]}^{2}}}\frac{\left|R\right|}{1-{\left|R\right|}^{2}}exp\left\{\frac{2kacos\theta }{\pi }P\int_{0}^{ka}\frac{xtan^{-1}\left[-\;\frac{J_{1}\left(x\right)}{N_{1}\left(x\right)}\right]}{\left[x^{2}-{\left(kasin\theta \right)}^{2}\right]\sqrt{\left[x^{2}+{\left(ka\right)}^{2}\right]}}dx\right\}$$

where *|R|* is the reflection coefficient of the dominant mode, given by:

(5) $$\left|R\right|=\;exp\left\{-\frac{2ka}{\pi }\int_{0}^{ka}\frac{tan^{-1}\left[-\frac{J_{1}\left(x\right)}{N_{1}\left(x\right)}\right]}{x\sqrt{\left[{\left(ka\right)}^{2}-x^{2}\right]}}dx\right\}\;,$$

*J_1_* and *N_1_* are the first-order cylinder functions, *k = 2π/λ* is the propagation constant of sound waves in free space, and *P* is the notation used to indicate the Cauchy principal value.

## 3. Experimental Setup

**Figure 1:**
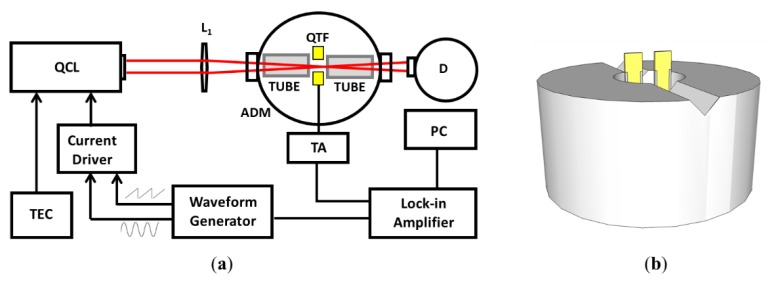
(**a**) Schematic of the quartz-enhanced photoacoustic spectroscopy (QEPAS) trace gas sensor system used. The laser beam is focused in the acoustic detection module (ADM) by means of a lens L_1_. TEC: temperature controller. PC: personal computer. TA: transimpedance amplifier. D: optical detector. (**b**) Sketch of the hollow metallic cylinder: the quartz tuning fork (QTF) is positioned in the center and tubes (not shown in the picture) are located on the V-groove.

In the on-beam spectrophone configuration, the QTF is positioned between the tubes. The geometrical parameters of the tubes must be properly chosen in order to ensure a sound amplification that is as high as possible. The scope of this work is to use the theoretical model proposed in the previous section to predict the optimal geometrical parameters of resonators when coupled with a QTF with a large prong spacing. A custom-made QTF with *s* =1.5 mm, having a prong length of 9.4 mm and a prong thickness of 2.0 mm, similar to the QTF-S15 presented in Patimisco et al. [6], has been employed. This QTF exhibits a resonance frequency of 15,801.6 Hz and a quality factor of 15,400 in air at atmospheric pressure. This QTF was implemented in the QEPAS setup that is schematically depicted in Figure 1.

A single-mode, continuous-wave quantum cascade laser (QCL) emitting at 7.8 μm was employed as the excitation source to generate pressure waves via the photoacoustic effect, targeting a water vapor absorption line at 1297.19 cm^−1^ with a line strength of 3.6 × 10^−22^ cm/molecule [18]. At atmospheric pressure, the selected water vapor line is well isolated from other spectral lines of molecules present in standard air. The laser beam was focused between the QTF prongs, 2 mm below the prongs top, using a ZnSe lens with a focal length of 50 mm. The QTF was soldered in an acoustic cell and positioned in the center of a hollow metallic cylinder with a V-groove on the top, properly designed to easily accommodate and fix resonator tubes with different lengths and diameters (see Figure 1b). The gas cell was filled with standard air at atmospheric pressure. The water vapor concentration was kept fixed at 1.7% using a Nafion humidifier and monitored by an external hygrometer. The QCL scanned the selected H_2_O absorption line using a slow ramp applied to the current driver. The sensor operated using wavelength modulation and 2f-detection: the laser current was modulated at half of the QTF resonance frequency and the QTF signal was demodulated using a lock-in amplifier at the resonance frequency. Once the spectral scan was recorded, the peak value of the QTF signal corresponding to the water absorption peak was extracted. 

## 4. Results and Discussion

In an on-beam QEPAS spectrophone, each tube is located close to the QTF, perpendicular to its surface, and their dimensions are correlated with the sound wavelength: this results in tubes with lengths in the millimeter range separated by much less than 1 mm. Hence, the tubes can be considered acoustically coupled to each other and the QTF as a probe for the acoustic vibration excited inside the tubes. In other words, the point-sized sound waves source (generated by the photoacoustic effect in the gas) is located between the tubes: the sound wave isotropically propagates inside the two tubes, becomes reflected at one end, and then propagates back to the other end of tube, where one part is returned back inside the tube in reflected waves and one part propagates in the other tube until a standing wave pattern is formed. The tubes’ geometrical parameters influencing the optical coupling are the internal diameter *ID* and the length of the two tubes together with the spacing between the tube and the QTF. Due to the ease of processing, low cost, and high quality of the inner surface, metal microresonator tubes have been chosen for this work. Six different tubes with internal diameters *ID* = 1.36 mm, 1.41 mm, 1.59 mm, 1.83 mm, 2.06 mm, 2.31 mm, and 2.41 mm were investigated at different lengths. By using OEC, the optimal tube length could be estimated. The OEC has been applied to all QTFs used so far for QEPAS sensing to predict the optimal tube length *l_th_* maximizing the sensor performance. In Table 1, the tube lengths predicted using Equation (3) were compared with those experimentally found to maximize the QTF signal (*l_exp_*).

From this comparison, it is evident that the OEC predicts the optimal tube length well with an uncertainty of less than 10%. Based on Equation (3), the optimal tube length for the QTF used in this work should linearly decrease from 9.8 mm to 9.0 mm when the tube diameter ranges from 1.3 mm to 2.4 mm. 

**Figure 2:**
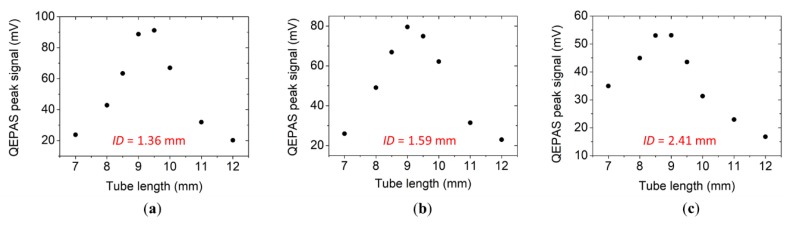
QEPAS peak signals measured with three different spectrophones employing acoustic resonator tubes with an *ID* = 1.41 mm (**a**), 1.59 mm (**b**), and 2.06 mm (**c**) as a function of the tube length.

In Figure 2a–c, the QTF peak signal measured with the setup shown in Figure 1a is plotted as a function of the tube length for three representative tube diameters. In all cases, the tubes were placed 150 µm away from the QTF surface. Each point is the mean value of peak values found using at least 20 spectral scans. The fluctuations of the peak value are comparable to a 1-σ off-resonance noise value (σ—standard deviation), estimated by tuning the laser far from the water absorption peak.

A *l_exp_* of 9.5 mm was measured for *ID* = 1.36 mm and *ID* = 1.41 mm, while a *l_exp_* = 9.00 mm was found for larger diameters. These results show that the optimal tube length weakly varied with the internal diameter and were in good agreement with the values predicted by the theoretical model, considering the precision on the tubes cut to 500 µm.

**Figure 3:**
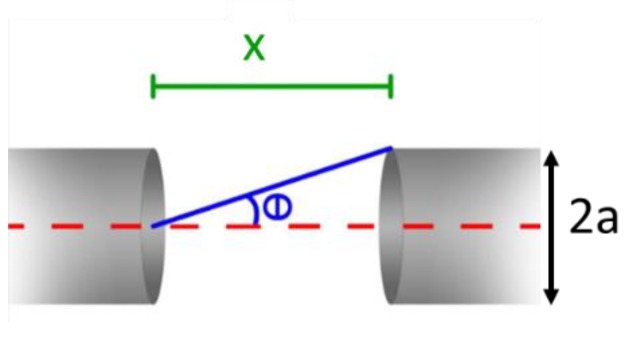
Schematic of two tubes separated by a distance *x*. The sound field included within the cone having height *x*, vertex angle *θ*, and base 4πa^2^ is supposed to couple with the opposite tube.

The second geometric parameter to be optimized was the distance between the two tubes, which was much smaller than their length. For this reason, the two tubes can be considered as a 1D single resonator. According to the theoretical model proposed, the optimal distance between the tubes is the one maximizing the sound energy transfer from one tube to the other. Geometrically, the transferred energy corresponds to the energy propagating within the cone having a vertex in the center of the open end of one tube and a base at the outer section of the opposite tube, as shown in Figure 3.

**Figure 4:**
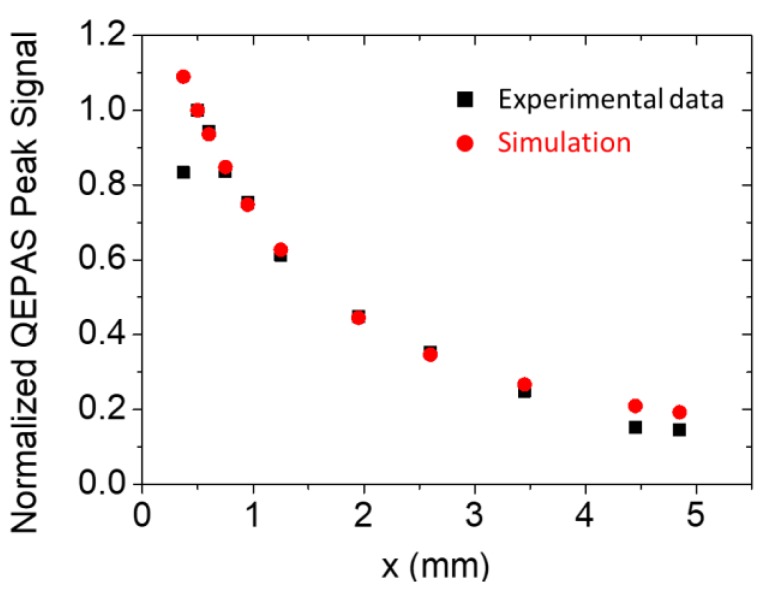
Normalized QEPAS Peak signal as a function of the distance between the two tubes (■), together with the theoretical simulation (●). Both experimental and theoretical values are normalized to the respective value measured at *x* = 550 µm.

With a fixed tube diameter 2*a*, the vertex angle of the cone scales with the tube distance *x* is given as *θ*(*x*) *= tan*^−1^*(a/x*). Hence, the amount of transmitted energy can be estimated by integrating Equation (4) from *0* to *θ*(*x*). With tubes having a length of 9.5 mm and internal diameter of 1.59 mm, the experimental results and theoretical data are plotted in Figure 4 as a function of the distance *x*. To compare the two trends, each set of data has been divided by the respective value obtained at *x* = 550 µm, which corresponds to the maximum of the experimental data.

For 550 µm ≤ *x* ≤ 4.8 mm, the theoretical model matched the experimental data, proving that the QTF acted as a perfect probe for the acoustic field intensity measurement in the area between the two tubes. The highest intensity QEPAS signal occurred at *x* = 550 µm, which corresponds to an optimal distance between the QTF surface and the tubes of 150 µm, considering a 250 µm crystal thickness. For a shorter distance, *x* = 400 µm, the QTF signal was reduced. This drop was not predicted by the theoretical model but can be explained by considering that when tubes are too close to the QTF, they generate a damping of QTF vibrations, causing an overall quality factor decreasing that negatively affects the QTF signal [19,20]. At distances larger than 5 mm, the sound energy transfer between the tubes was highly reduced; therefore, these distances are not feasible for QEPAS sensing.

**Figure 5:**
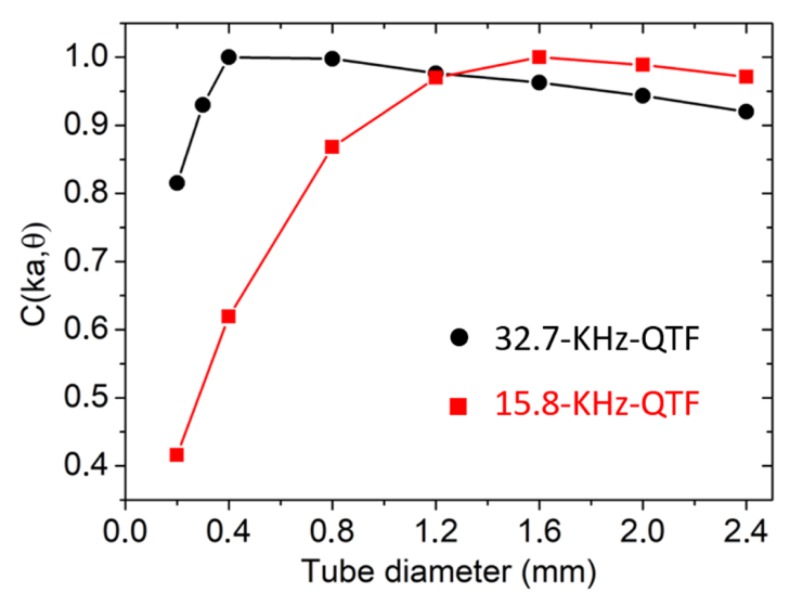
Normalized *C*(*ka,θ*) calculated as a function of tube diameter by using Equations (4) and (6), for the 15.8-kHz QTF and the standard 32.7-kHz QTF.

The theoretical model can also be used to predict the optimal tube radius that maximizes the QTF signal. Placing both tubes at a distance of 150 µm away from the QTF, the vertex angle of the cone will scale according to *θ*(*a*) *= tan*^−1^(*a/*0.55) as a function of the tube radius *a* (in millimeters). However, if the prong spacing becomes lower than the tube diameter, part of the sound field exiting from the tube will be shielded by the QTF surface. As a consequence, the effective angle *θ* will become *s/*0.55, for all *a* ≥ *s/*2. The amount of energy transfer can be estimated as:

(6) $$C\left(ka,\theta \right)=2\int_{0}^{\theta \left(a\right)}G\left(ka,\theta \right)d\theta$$

considering the shielding effect due to the prong’s surface when *a* ≥ *s/*2. The results of the simulation are shown in Figure 5 for the QTF under investigation and for the standard 32.7-kHz QTF (each curve has been normalized to the related highest value). The model proposed by Levine et al. [17] requires that *ka* << 1. For a 15.8-kHz QTF, *ka* = 0.22, while for the standard 32.7-kHz QTF, it is equal to 0.18. Hence, it is safe to use the theoretical model for both QTFs.

**Figure 6:**
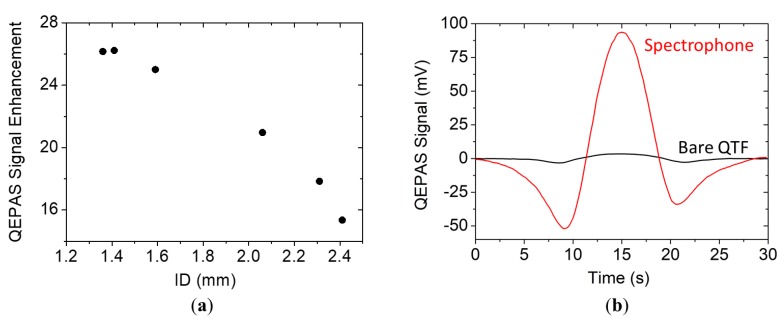
(**a**) Signal enhancement of the spectrophone signal with respect to the bare QTF signal as a function of the tubes’ internal diameter when the optimal tube length was used for each *ID*. (**b**) QEPAS spectral scan of water absorption line acquired with the bare QTF (black solid line) and with a spectrophone composed of the QTF and a pair of resonator tubes having a length of 9.5 mm and internal diameter of 1.41 mm, both positioned 150 μm away from the QTF surface (red solid line).

As predicted, the optimal tube diameter depended on the sound wavelength and the prong spacing. For a standard 32.7-kHz QTF, the model predicted optical tube diameters in the range 400–800 µm, in agreement with the values found in Dong et al. [3]. Moving to 15.8 kHz and increasing the prong spacing up to 1.5 mm, the theoretical optical tube diameter fell in the range 1.4 mm ≤ *a* ≤ 1.8 mm. In Figure 6a, the QEPAS peak signal enhancement with respect to the bare QTF is reported as a function of the internal diameter for tubes having *l_exp_* = 9.5 mm for *ID* = 1.36 mm and 1.41 mm and *l_exp_* = 9.0 mm for all other IDs.

The experimental results show that the largest signal enhancement was obtained for a tube diameter of 1.41 mm, in agreement with the theoretical prediction. Differently from the simulation, the QTF signal decreased rapidly when tubes with diameters greater than the optimal one were used. The model failed to predict the experimental trend when *a* » *s/*2, since in this case, in addition to pressure wave shielding, the prongs’ surfaces distorts the standing wave pattern, thereby altering the resonance conditions and the sound waves enhancement. Tube diameters lower than 1.38 mm cannot be used to validate the theoretical model since photothermal noise on the QTF signal arises due to a portion of the laser beam touching parts of the spectrophone. The QEPAS scan of the water absorption line measured with the optimal spectrophone (*l* = 9.0 mm and *ID* = 1.41 mm) is shown in Figure 6b, together with the QEPAS scan acquired with the bare QTF. The QEPAS peak signal was 93.6 mV, corresponding to a signal enhancement of 26 with respect to the bare QTF, with a comparable noise level, demonstrating that good sound amplification levels can also be achieved with a QTF with a large prong spacing that is comparable to the best result obtained with the standard 32.7-kHz QTF [3], with the advantage of having halved the resonant frequency and exploited a prong spacing that was 5 times larger.

## 5. Conclusions

In this work, we proposed a theoretical model that was able to predict the optimal tube geometries for a QEPAS spectrophone. An open-end correction due to an impedance mismatch between the acoustic field inside the resonator and outside it must be introduced to predict the optimal length of each tube. The open-end correction depends only on the tube radius. The optimal tube radius can be estimated by considering the acoustic coupling between two tubes, i.e., the amount of the acoustic field transferred from one tube to the other one. The optimal tube radius strongly depends on the sound wavelength and prong spacing. The theoretical model was validated by testing spectrophones implementing a QTF with a 15.8-kHz resonance frequency and a prong spacing of 1.5 mm and tubes with different dimensions. These spectrophones were employed in a QEPAS sensor for atmospheric water vapor detection. Tubes having a length of 9.0 mm and an internal diameter of 1.41 mm, positioned 150 µm away from the QTF surface, provided the best performance in terms of QEPAS signal enhancement, in good agreement with the theoretical prediction. With these conditions, a 26-fold increase of the QEPAS signal with respect to the bare QTF was achieved.

## Figures and Tables

**Table 1:** Best geometry parameters of dual-tube spectrophones realized for four different QTFs operating at different frequencies: the prongs’ spacing (*s*), the internal diameter of the tube (*ID*), the sound half wavelength (λ/2), and the theoretical (*l_th_*) and experimental (*l_exp_*) optimal tube lengths.

QTF Frequency	Prong Spacing *s* (mm)	*ID* (mm)	*λ/2* (mm)	*l_th_* (mm)	*l_exp_* (mm)	Ref.
32.7 kHz	0.3	0.60	5.25	4.7	4.4	[3]
7.2 kHz	0.8	1.30	23.9	22.8	23.0	[10]
25.4 kHz	1.0	1.52	6.8	5.5	5.3	[5]
12.4 kHz	0.8	1.59	13.8	12.4	12.4	[6]
